# Supplementary material for: Virtual substrate method for nanomaterials characterization
Source: Nat Commun. 2017 May 26;8:15629. doi: 10.1038/ncomms15629 (PMC5458549; doi:10.1038/ncomms15629)
Supplement: Supplementary Information — Supplementary Figures and Supplementary Notes. [file ncomms15629-s1.pdf]

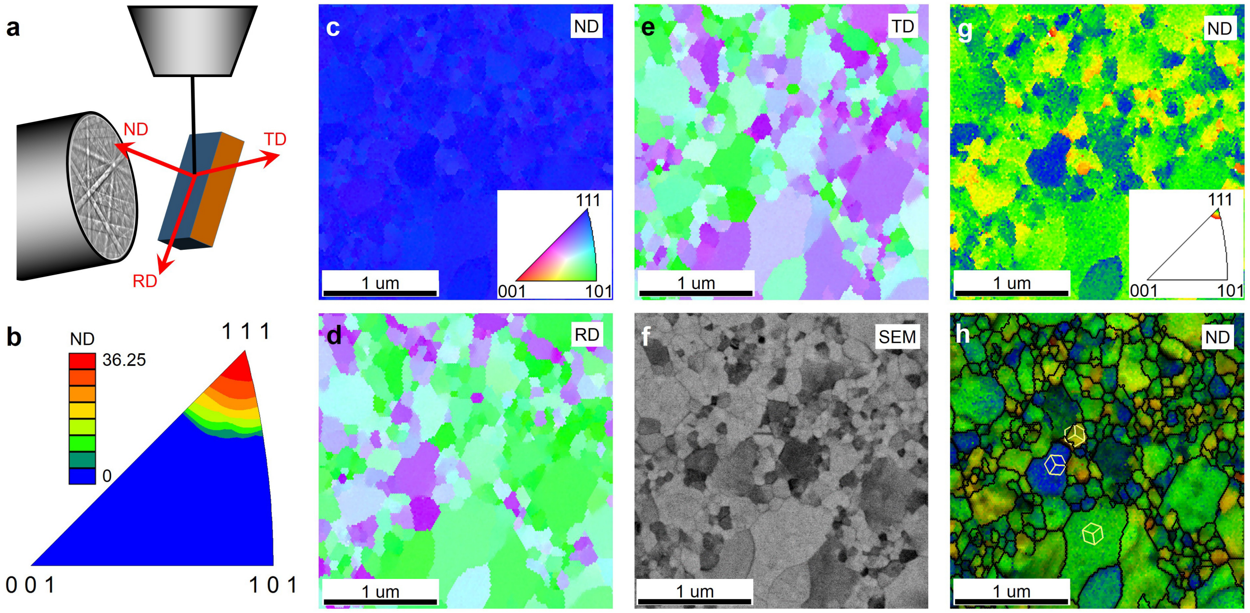

Supplementary Figure 1. Polycrystalline gold film substrate studied by electron backscatter diffraction (EBSD). a, Geometry of the sample setup for the EBSD measurement and the definition of the normal direction (ND), rolling direction (RD) and transverse direction (TD) of the sample. **b**, Misorientation-axis distributions in ND of the polycrystalline gold substrate in the virtual substrate method. EBSD maps for **c**, ND, **d**, RD and **e**, TD and **f**, SEM image of the same region of the sample. The colour maps of the EBSD for different directions are the same, as shown in the inset of **c**. The low-angle boundaries ($\theta < 5°$) of the same area of the gold film are presented in **d**, with a colour map as an inset. The corresponding EBSD image quality map, a greyscale image in transparent scale, covering the EBSD map of the low-angle boundaries ($\theta < 5°$), is shown in **f**. The high-angle boundaries ($\theta > 5°$) are also included as thick black lines.

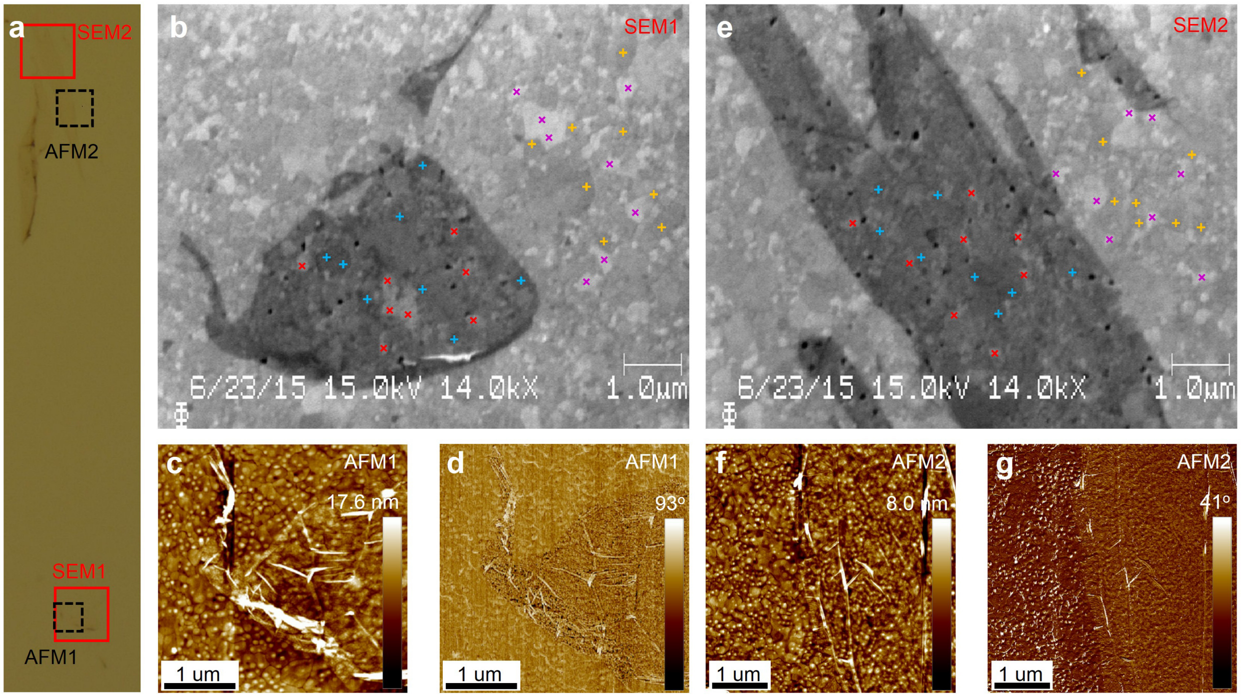

Supplementary Figure 2. Mono- and bilayer graphene on gold substrates characterized by optical microscopy, SEM and AFM. a, Optical microscopy image of mono- and bilayer graphene on a gold film. The layer number of graphene sheets was determined by Raman spectroscopy. The regions covered with monolayer graphene marked as SEM1 and AFM1 were characterized by **b**, SEM, **c**, AFM and **d**, AFM phase images. The regions covered with bilayer graphene denoted as SEM2 and AFM2 were characterized by **e**, SEM, **f**, AFM and **g**, AFM phase images. The incident beam positions in the eight groups of virtual substrate measurements are also presented in the SEM image as pink diagonal crosses, yellow crosses, red diagonal crosses and blue crosses for measurements performed on bare Au(A) (bright area in SEM), bare Au(B) (dark area in SEM), graphene on Au(A) and graphene on Au(B), respectively.

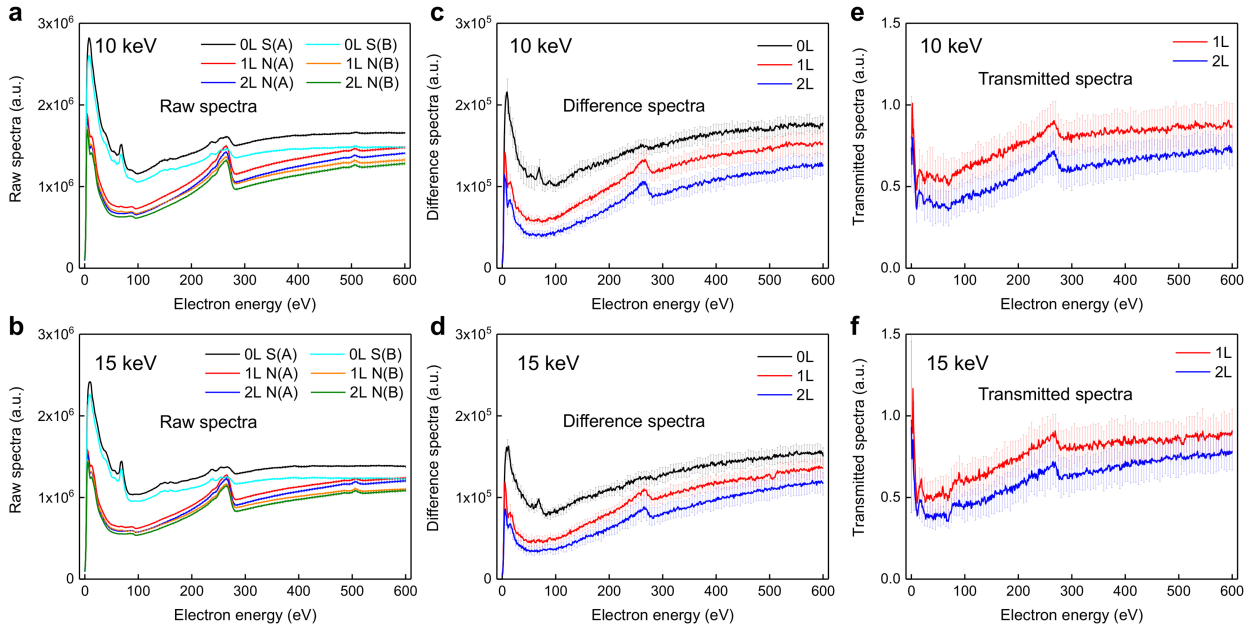

Supplementary Figure 3. Process of virtual substrate measurements on mono- and bilayer graphene/gold samples. a, **b**, raw spectra, **c**, **d**, difference spectra and **e**, **f**, transmitted spectra obtained from eight independent groups of virtual substrate measurements in the energy range from 0 to 600 eV with an energy step of 1 eV for mono- or bilayer graphene on gold substrates with incident electron energies of 10 keV (top) and 15 keV (bottom). The standard deviations for the eight group measurements are presented as error bars at intervals of 5 eV.

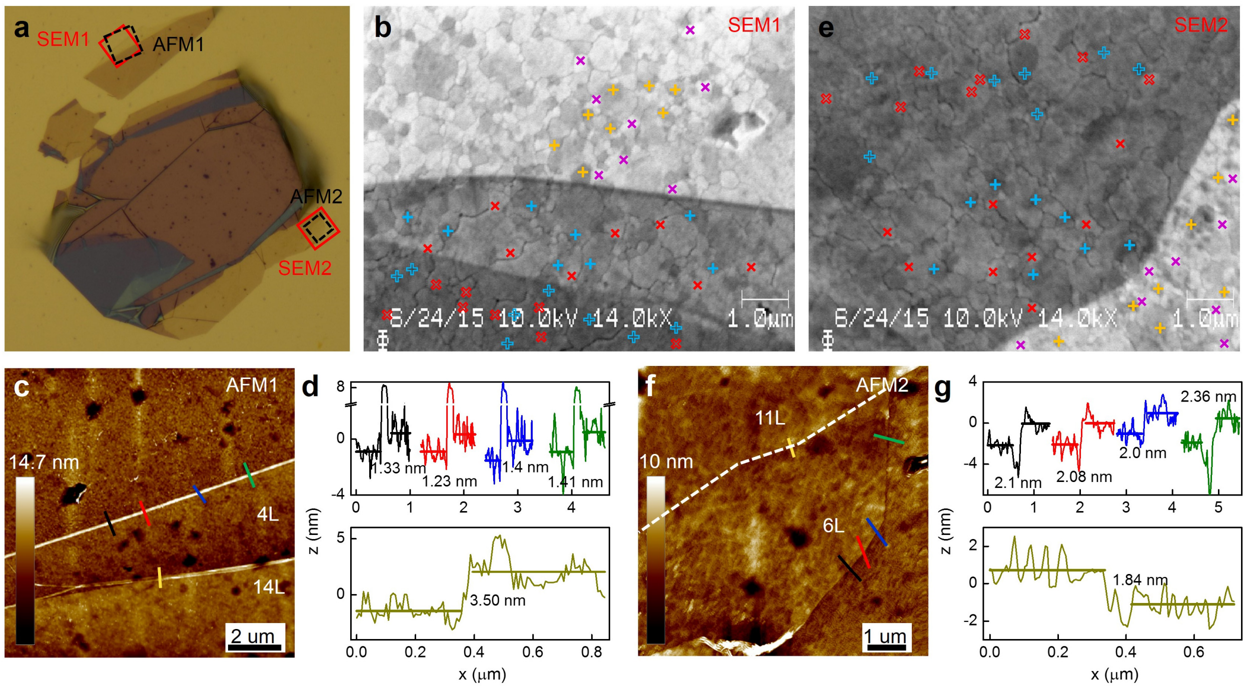

Supplementary Figure 4. Four-, six-, eleven- and fourteen-layer graphene on a gold substrate characterized by optical microscopy, SEM and AFM. The number of layers in the graphene was estimated by cross-sectional AFM. Raman spectroscopy was also employed to confirm the number of layers in four-layer graphene. **a**, Optical microscopy image of few-layer graphene sheets on a gold film. The regions with four- and fourteen-layer graphene marked as SEM1 and AFM1 were observed by **b,** SEM and **c,** AFM. Regions with six- and eleven-layer graphene marked as SEM2 and AFM2 were identified by **e**, SEM and **f**, AFM. The incident beam positions in the eight groups of virtual substrate measurements are also presented in the SEM image as pink diagonal crosses, yellow crosses, red diagonal crosses, blue crosses, red diagonal open crosses and blue open crosses for measurements performed on bare Au(A) (bright area in SEM), bare Au(B) (dark area in SEM), four-layer graphene (SEM1) and six-layer graphene (SEM2) on Au(A), four- and six-layer graphene on Au(B), eleven-layer graphene (SEM2) and fourteen-layer graphene (SEM1) on Au(A) and eleven- and fourteen-layer graphene on Au(B), respectively. The cross-sectional

profile near the steps in the graphene samples, marked as colored lines in the AFM images, are presented in **d** and **g** along with the line fits of the layer heights.

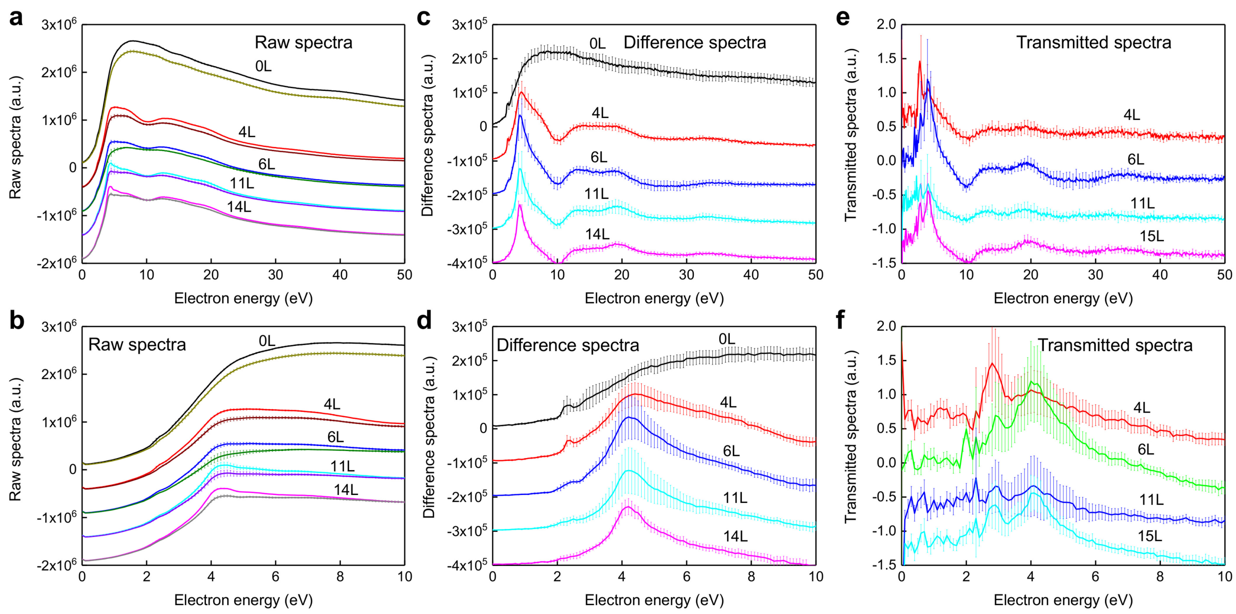

Supplementary Figure 5. Virtual substrate measurements of few-layer graphene/gold samples in the low-energy range. a, **b**, raw spectra, **c**, **d**, difference spectra and **e**, **f**, transmission spectra obtained from eight independent groups of virtual substrate measurements in the energy range from 0 to 50 eV (top) or 10 eV (bottom) with 0.1 eV energy steps for four-, six-, eleven- and fourteen-layer graphene on a gold substrate with an incident electron energy of 10 keV. The standard deviations for the eight group measurements are presented as error bars at intervals of 0.5 and 0.1 eV for measurements in energy ranges up to 50 and 10 eV, respectively.

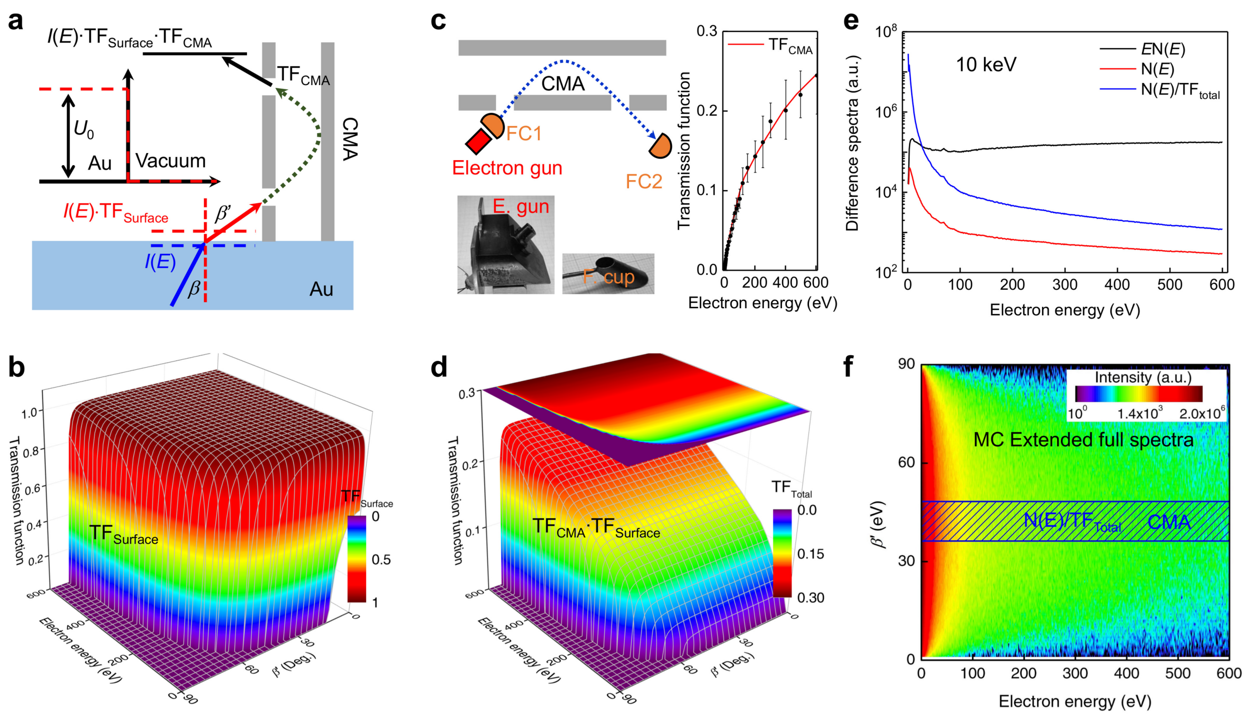

Supplementary Figure 6. Process to calculate extended full spectra from the difference spectra of bare substrates. **a**, Schematic diagram of the method used to restore the realistic measured spectra to the moment before entering the CMA detector by considering the transmission function of the CMA detector, and the moment before being emitted from the sample surface by adding the contribution from surface potential barrier estimated using a square barrier model (inset). **b**, The transmission function of surface potential barrier, $TF_{Surface}$, for a gold/vacuum interface calculated as functions of electron energy and electron emission angle. **c**, Schematic diagram of the measurement of the transmission function of CMA, TF_{CMA}. Measured values are shown as black circles with error bars representing experimental uncertainty. Fitting curves from 1 eV to 5 keV are shown as red lines. Inset are photographs of the mini electron gun and Faraday cup used in the measurement. Image reprinted with permission from ref. 15, Surface Analysis Society of Japan. **d**, The total transmission function, TF_{Total}, calculated by multiplying $TF_{Surface}$ and TF_{CMA}, plotted as functions of electron energy and electron emission angle. **e**, Difference spectrum,

EN(E), (black line) obtained for a bare substrate in a virtual substrate measurement with incident electron energy of 10 keV, the corresponding N(E) spectrum (red line) and the restored realistic difference spectrum, N(E)/TF_{Total}, (blue line) in the energy range from 1 to 600 eV. **f**, The extended full spectra, restored emitted electron energy and moving direction distribution expanded from the restored realistic difference spectrum in **e** using a MC simulation program.

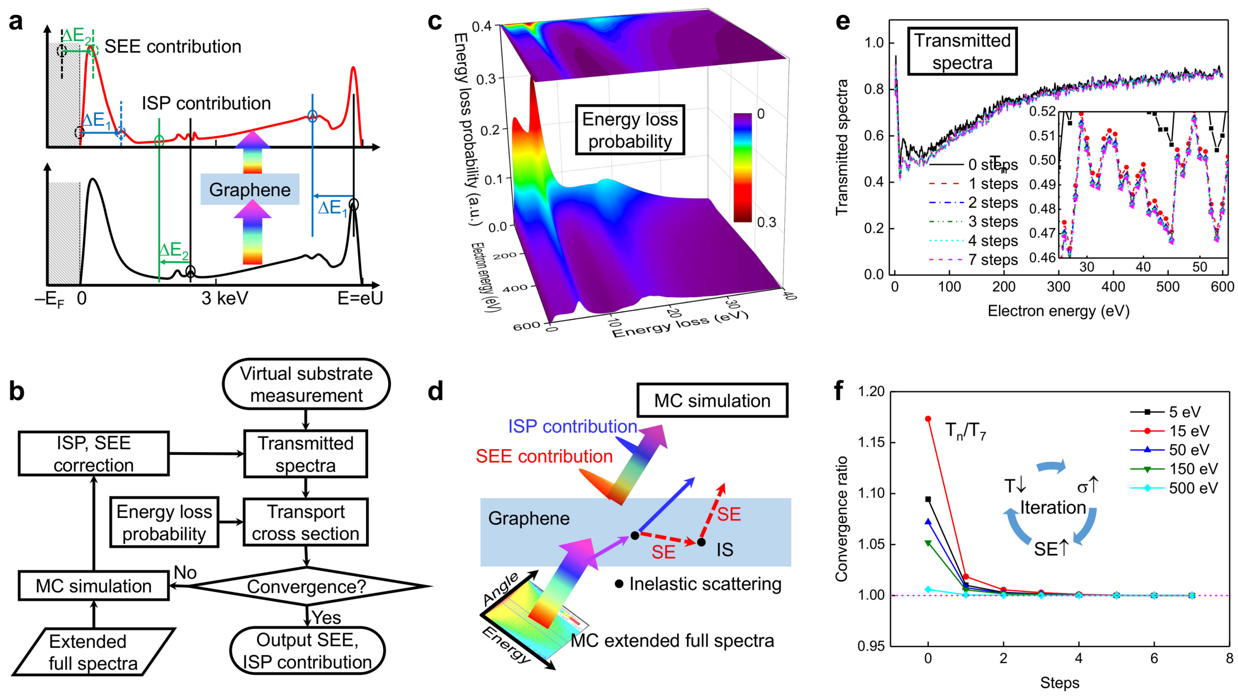

Supplementary Figure 7. Process to remove the ISP and SEE contributions from the virtual substrate measurements. **a**, Schematic diagram of how ISP and SEE contribute to the transmitted spectra in a virtual substrate measurement. **b**, A flow chart of the self-adaptive iterative MC simulation program used to remove ISP and SEE contributions from the virtual substrate measurements. **c**, Energy loss probability calculated based on the dielectric response theory employed in the self-adaptive iterative MC simulation program. **d**, Schematic diagram of the MC simulation program used in the self-adaptive iterative MC simulation program. **e**, Updating process of the simulated transmitted spectra in the self-adaptive iterative MC simulation program. Inset is the energy range from 25 to 55 eV. **f**, The convergence ratio normalized by the final transmitted spectrum (seventh transmitted spectrum, T_7). Inset is the relationships between the intensity of electron transmission, T, the transport cross section, σ, and the intensity of ISP+SEE contributions, marked as SE for short.

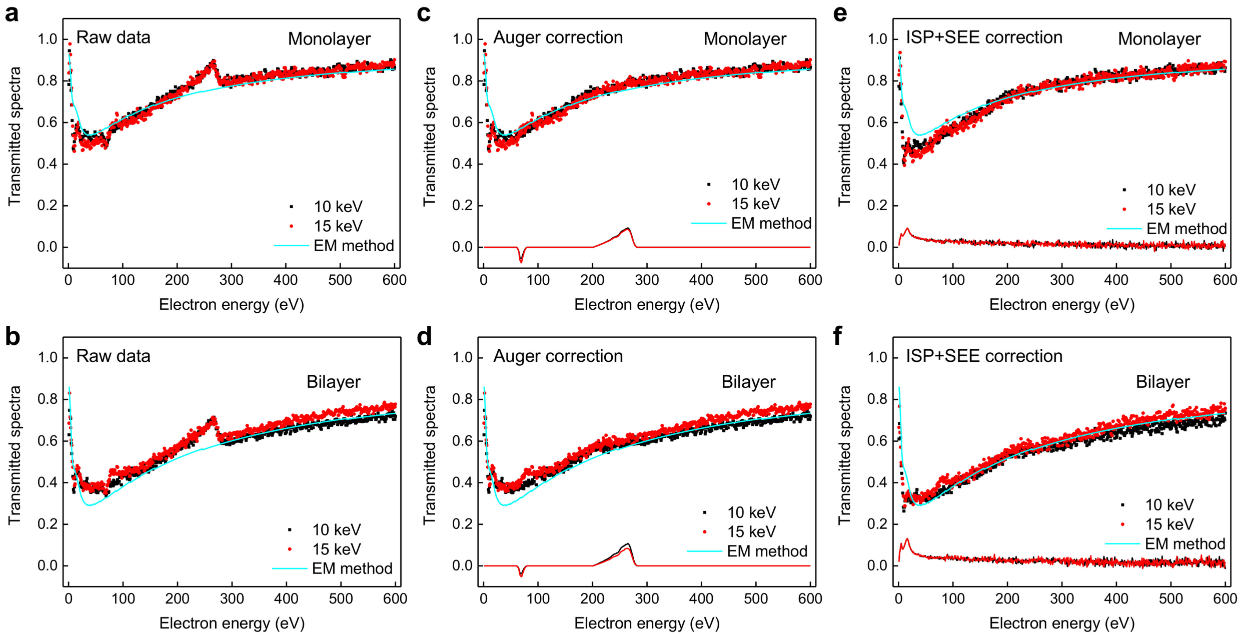

Supplementary Figure 8. The Auger electron, ISP and SEE contributions used to modify the transmitted spectra. Transmitted spectra for **a**, monolayer graphene, and **b**, bilayer graphene with incident electron energies of 10 and 15 keV determined according to the virtual substrate method together with values calculated by the EM method. The Auger contributions in transmitted spectra for **c**, monolayer graphene, and **d**, bilayer graphene together with absolute values of these corrections in transmitted spectra. The ISP+SEE contributions in transmitted spectra for **e**, monolayer graphene, and **f**, bilayer graphene together with absolute values of these corrections in transmitted spectra.

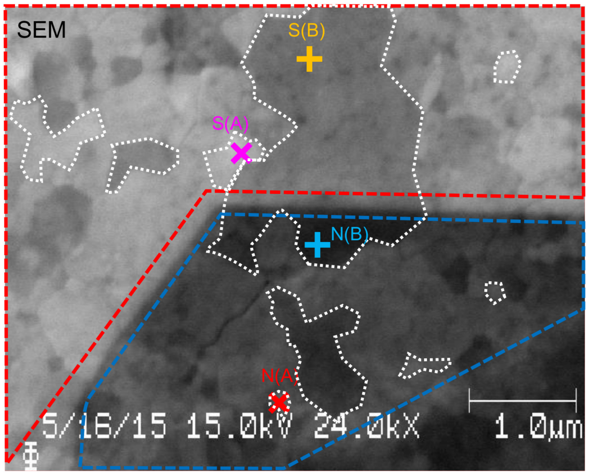

Supplementary Figure 9. The simplest way to select measurement points. SEM image of a graphene/gold system. The bare gold substrate and graphene-covered regions are circled by red and blue dashed lines, respectively. The pink diagonal, yellow, red diagonal and blue crosses represent the selected measurement points marked as S(A), S(B), N(A) and N(B), respectively. Some of the dark and bright areas are highlighted by white dotted lines.

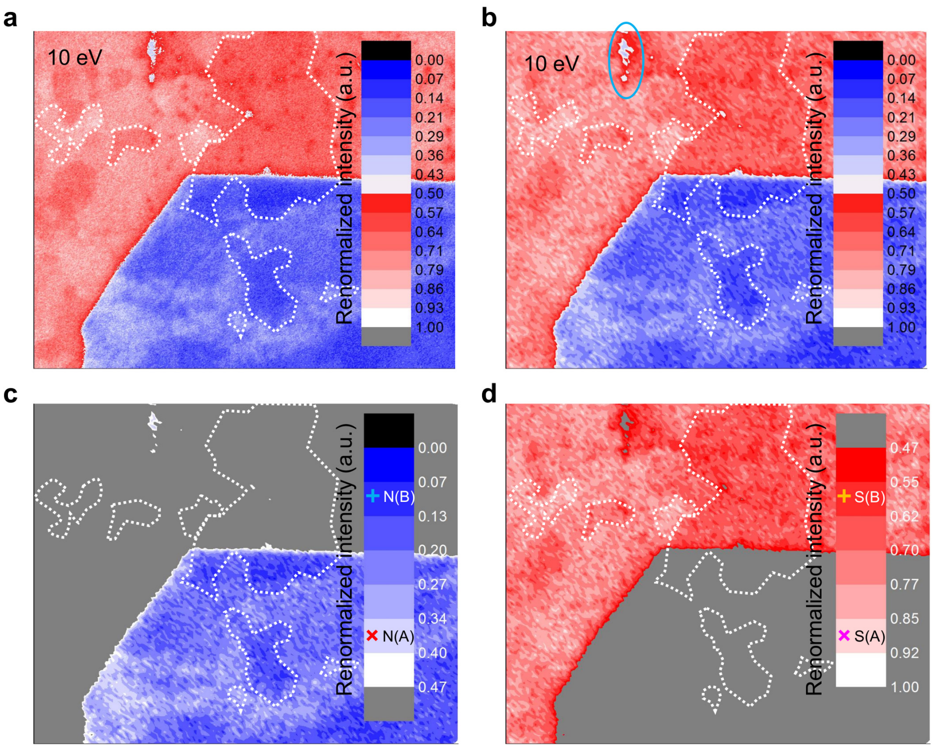

Supplementary Figure 10. Renormalization and smoothing of the SEM image. a, The renormalized SEM image (only 10 eV electrons) where the raw intensities are subtracted by the minimum intensity value and then divided into 14 levels according to their intensities. **b**, The renormalized SEM image obtained by a combination of the renormalization procedure and smoothing operation. The abnormal pixels in the SEM image are indicated by a blue circle. **c**, The renormalized SEM image for the graphene-covered region where the renormalization procedure is performed using only pixels in this region after removing the abnormal pixels. **d**, The renormalized SEM image for the gold substrate region after the same treatment as in **c**. The pink diagonal, yellow, red diagonal and blue crosses indicate that the possible measurement points S(A), S(B), N(A) and N(B), respectively, can be selected at any region where the same colour is shown corresponding to the position of these cross marks on the colour map.

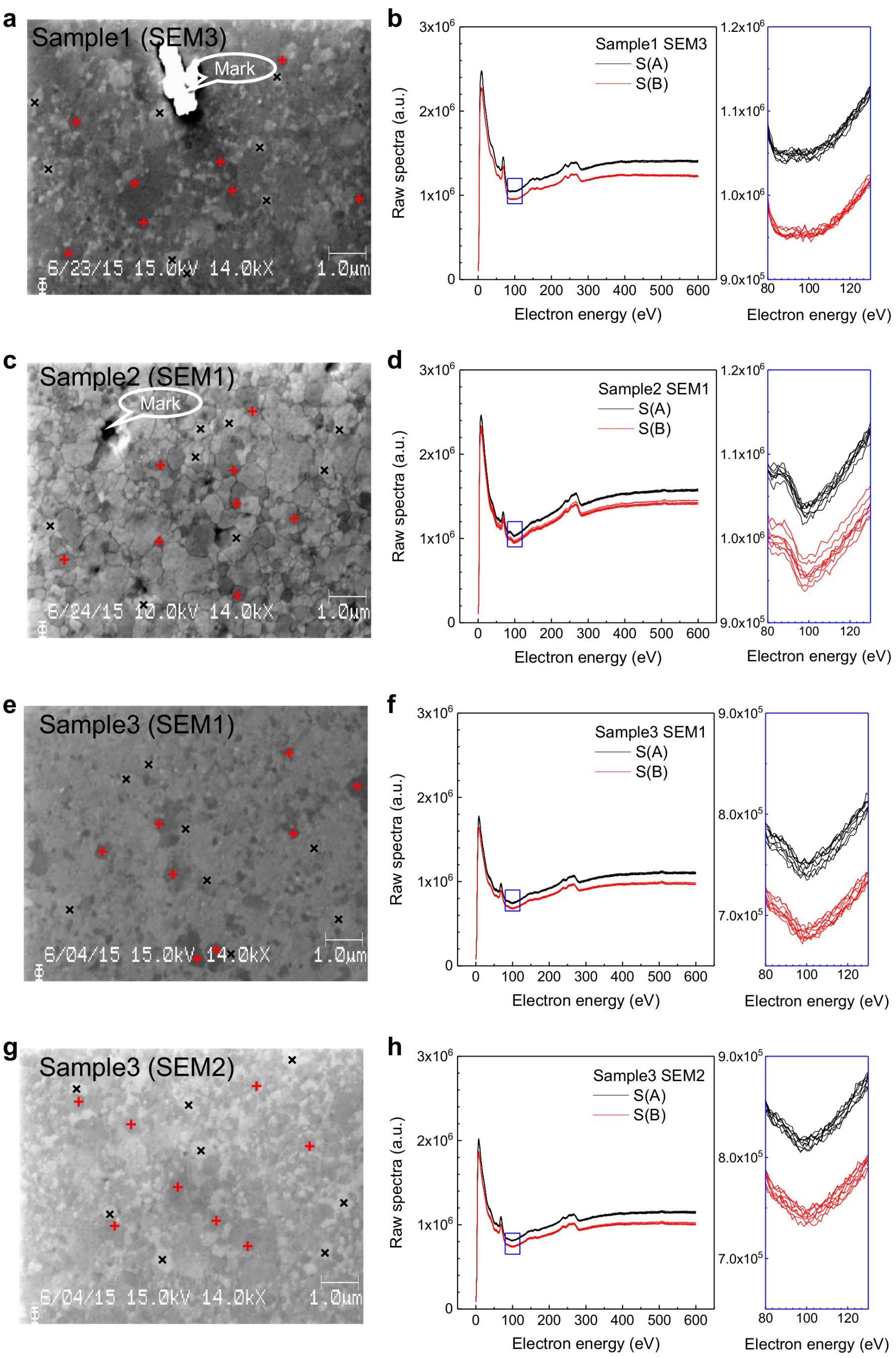

Supplementary Figure 11. Pre-testing of a gold substrate before transfer of a graphene layer. Four pre-testing procedures are performed for three different samples denoted as Sample 1, Sample 2 and Sample 3. Two pre-testing procedures are performed on Sample 3 in two different areas roughly 2 mm apart. The SEM images corresponding to the four pre-testing procedures are shown

in **a**, **c, e,** and **g** with the measurement points marked as black diagonal crosses (bright areas) and red crosses (dark areas). The resulting raw spectra for these four pre-testing procedures are provided in **b**, **d, f,** and **h** with an additional enlarged picture on the right of the region encircled by a blue line. In every pre-testing procedure, up to 16 raw spectra are measured; eight of bright areas and eight of dark areas.

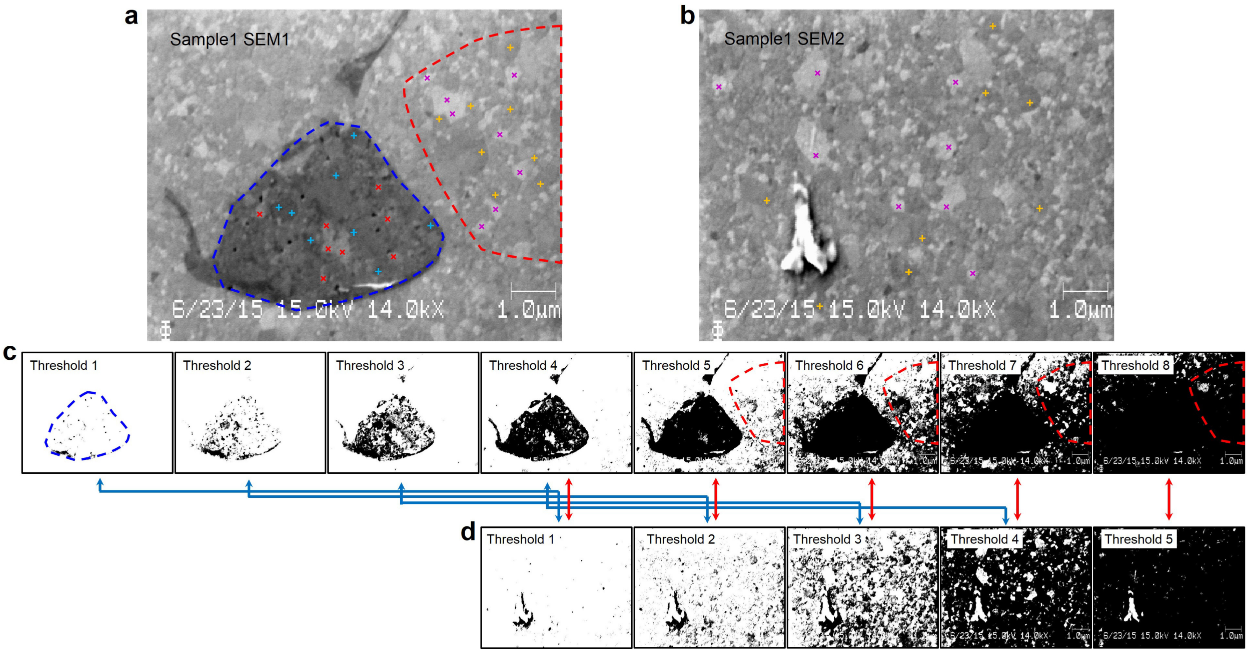

Supplementary Figure 12. Selecting measurement points in a practical virtual substrate measurement. **a**, SEM image of graphene on top of a gold substrate (Sample 1, SEM1). The blue and red dashed circles represent the appointed areas for selecting measurement points in the graphene-covered and bare gold substrate regions, respectively. **b**, SEM image of the bare gold substrate (Sample 1, SEM2). The pink diagonal, yellow, red diagonal and blue crosses represent the selected measurement points marked as S(A), S(B), N(A) and N(B), respectively. **c**, A schematic diagram of the process for determining measurement points using our custom-made software corresponding to the sample shown in **a** (Sample 1, SEM1). **d**, The same process for the sample shown in **b** (Sample 1, SEM2).

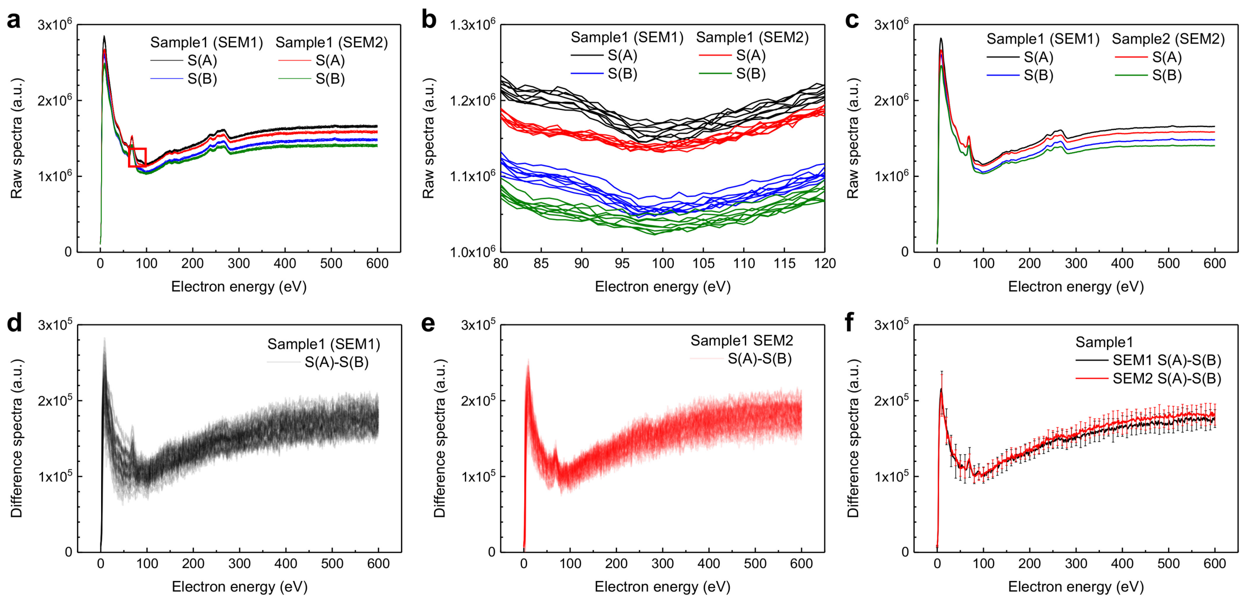

Supplementary Figure 13. Spectra measurement at selected points. a, Raw spectra measured at selected on the bare gold substrate region shown in Supplementary Figure 12a and b. For each type of selected measurement points, eight spectra were measured and are plotted together as lines of the same colour. Black and blue lines represent the measurements corresponding to the SEM image in Supplementary Figure 12a (Sample 1, SEM1), while the red and green lines represent the measurements corresponding to the SEM image in Supplementary Figure 12b (Sample 1, SEM2). **b**, Raw spectra plotted in the electron energy range of 80–120 eV (enlargement of the region indicated by a red rectangle in **a**). **c**, Averaged raw spectra from all eight measurements for each type of selected measurement points. **d**, The 64 possible difference spectra calculated from the raw spectra measured for the bare gold substrate (Sample 1, SEM1). **e**, The 64 possible difference spectra calculated from the raw spectra measured for the bare gold substrate (Sample 1, SEM2). **f**, The averaged difference spectra for the bare gold substrate from both SEM regions with error bars representing one standard deviation.

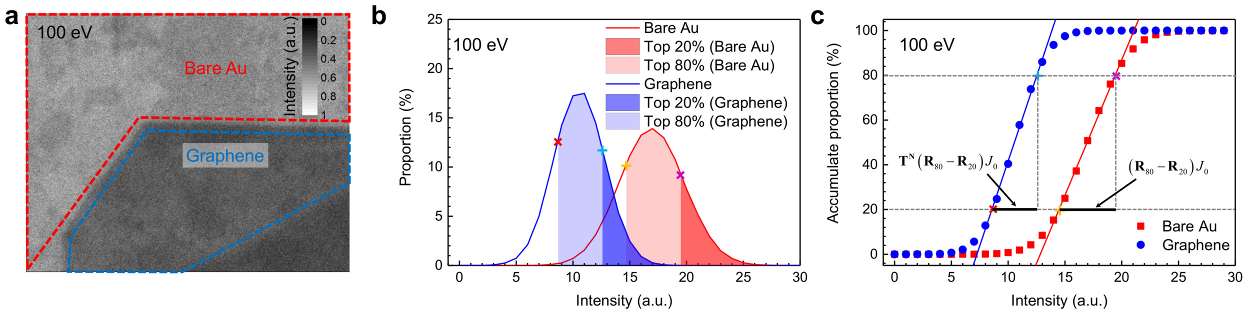

Supplementary Figure 14. Virtual substrate measurement without selecting measurement points. a, SEM image obtained from 100 ± 2 eV electrons. The bare gold substrate and graphene-covered regions are indicated by red and blue dashed lines, respectively. **b**, The proportion of pixels over 30 levels (relative intensity on the bottom *x*-axis) according to their intensities (electron count on the top *x*-axis) on the bare gold substrate and monolayer graphene-covered regions. The fictitious measurement points are marked as pink diagonal, yellow, red diagonal and blue crosses representing S(A), S(B), N(A) and N(B), respectively. **c**, The accumulated proportion of pixels on the bare gold substrate and graphene-covered regions plotted according to their intensities. The lines of best fit for the data points with an accumulated proportion value in the range of 15%–85% for the bare gold substrate and graphene-covered regions are presented. The deviation of intensities of the calculated accumulated proportions of 80% and 20% for the bare gold substrate and graphene-covered regions are indicated by thick black lines.

Supplementary Note 1. Separability correction of energy channels in virtual substrate measurement.

One problem that needs to be overcome in a virtual substrate method implemented by electron-based techniques is that the energy channels in the measurement are not separated because of the use of white electrons as a probe. Primary electrons suffer from an inelastic scattering process (ISP), and the accompanying SE emission (SEE) contributes to a virtual substrate measurement by generating undistinguished electrons with lower energies that can be confused with primary electrons of this energy. Here, a self-adaptive iterative MC simulation program was developed to solve this inverse problem by taking advantage of the well-developed MC technique to describe electron transport inside graphene sheets.

Before performing such a simulation, the electron energy and moving direction distribution of the probe spectrum (extended full spectrum) are required to reproduce the detailed processes of a probe spectrum traveling through graphene sheets as an input. Supplementary Fig. 6 shows how to determine such extended full spectra from the difference spectra for bare substrates. To calculate such extended full spectra, we need restore the difference spectra taken on a bare substrate back to the moment just before the electrons are emitted from the sample surface. To do this, transmission functions of the CMA detector, TF_{CMA}, and surface potential barrier, $TF_{Surface}$, are necessary, as shown in Supplementary Fig. 6a. To calculate $TF_{Surface}$, a simple square barrier model can be used, where the constant electronic potential U_0 in the interior of the gold substrate is defined as the sum of the kinetic energy at the Fermi level E_F (9.0 eV for gold) and the work function φ of gold (5.1 eV). The calculated $TF_{Surface}$ are presented as functions of electron energy and emission angle, β', in Supplementary Fig. 6b, where the electron energy refers to the vacuum level and β' is relative

to the surface normal. In the present work, TF_{CMA} was measured by putting a well-defined virtual source of electrons at the sample position to simulate real measurements, as illustrated in Supplementary Fig. 6c. The measured TF_{CMA} values with error bars representing the experimental uncertainty were plotted from 1 eV to 5 keV and extended to 0.1 eV using a theoretical model. Furthermore, the total transmission function, TF_{Total}, was determined by multiplying $TF_{Surface}$ and TF_{CMA} together, and has been plotted as functions of electron energy and β' in Supplementary Fig. 6d. Based on the determined TF_{Total}, the difference spectra for a bare substrate can be restored back to the moment just before being emitted from the surface, as shown in Supplementary Fig. 6e. However, the restored spectrum is only formed of those electrons whose emission angles are 42.3±6° according to the solid angle of the CMA detector. Other electrons with smaller or larger emission angles that may also contribute to the detected spectra after passing through the graphene sheet because the change of moving direction induced by the elastic scattering are missing. To include such contributions, MC simulation was employed to reconstruct this restored spectrum to the case with all possible emission angles, as illustrated in Supplementary Fig. 6f. According to the intensity of the restored difference spectra for a given energy, the intensity of the extended full spectra for every possible emission angle is magnified or contracted in equal proportion according to the angular distribution obtained by the MC simulation program while ensuring the sum of the electron number in this extended full spectrum within the detector angle (42.3±6°) equals that in the restored difference spectrum for every energy.

After obtaining the extended full spectra, we can use a self-adaptive iterative MC simulation program to remove the ISP and SEE contributions from a virtual substrate measurement in a self-consistent way, as illustrated in Supplementary Fig. 7. The ISP and SEE contributions involved in

a virtual substrate measurement are more important than those in traditional measurements because of the use of white electrons as a probe, as shown in Supplementary Fig. 7a. Both ISP and SEE contributions strongly depend on the number of graphene layers, as well as the substrate because of the diverse energy distribution of reflected electrons in the low energy range for different materials. A flow chart of the self-adaptive iterative MC simulation program used here is provided in Supplementary Fig. 7b. The algorithm is as follows:

(1) The virtual substrate measurement is performed without any theoretical correction for a given graphene/gold system and results in the initial transmitted spectra.

(2) According to the transmitted spectra, the corresponding transport cross section can be determined by adding the calculated energy loss probability based on the dielectric response theory as shown in Supplementary Fig. 7c with help of a MC program.

(3) Based on this determined transport cross section as well as extended full spectra, the MC simulation program as shown in Supplementary Fig. 7d for electrons passing through graphene layers under same configuration as in the experimental measurement is performed to determine the ISP and SEE contributions in the form of a correction in transmitted spectra.

(4) Determined ISP and SEE contributions are added into the previous transmitted spectra and result in new transmitted spectra, as shown in Supplementary Fig. 7e.

(5) Repeat steps 2, 3 and 4 for the next iteration. Successive iterations generate decreasing values of deviations of transmitted spectra or corresponding transport cross section between two contiguous iterations, as shown in Supplementary Fig. 7f.

It should be noted that a smaller transmission probability of electrons (larger transport cross section) leads to larger ISP and SEE contributions, which then affect the transmission with a decrease in probability, as shown in the inset of Supplementary Fig. 7f. This self-adaptive law guarantees the convergence of the iterative MC simulation program to determine the ISP and SEE contributions in a self-consistent manner.

Supplementary Note 2. The criteria for selecting measurements points when using polycrystalline gold substrate in virtual substrate measurement.

Precise virtual substrate measurement is very sensitive to the crystallographic orientations of the gold grains with and without covering nano-overlayer. Large relative orientations (more than 1° from EBSD study) of the gold grains in the measurement will lead to different intensities of reflected SE spectra from the gold substrates (white electrons) (~1% relative error), leading to inaccurate difference spectra $J_{\Delta S}$ and $J_{\Delta N}$ and inaccurate transmitted spectra $T_{\Delta N/\Delta S}$. In fact, besides the relative orientations of the gold grains, other differences between the gold substrates such as the different crystal quality and different surface morphology will also lower the accuracy of transmitted spectra because they results in inconsistent white electrons with and without the covering nano-overlayer. According to the definition of the virtual substrate method, this technique is not strict with the concrete parameters of gold grains, only with the reflected SE spectra produced from these gold grains (white electrons). Therefore, instead of estimating the similarity of gold grains by their various quantifiable parameters, we can directly evaluate the sum of their parameters from white electrons they produce. Whether or not the gold grains produce the same reflected SE spectra under the same experimental conditions is the best judge for the reliability of a substrate in a virtual substrate measurement. In this case, the accuracy of results from one virtual substrate measurement can be confirmed by comparing the spectra produced by the selected gold grains before transfer of the graphene layer. In practical application, once the same reflected SE spectra are achieved in two gold grains before transfer of the graphene layer, the reflected SE spectra will remain the same after transfer of a graphene layer onto one of them. Therefore, the best index to evaluate that two different gold grains are equivalent in a virtual substrate measurement is the consistency of the reflected SE spectra produced by these two different gold

grains. Once we find a series of measurement points on the surface of a substrate that produce the same reflected SE spectra, the virtual substrate measurement can be performed with high precision after transfer of a graphene layer to cover half of the measurement points.

Now, the question of how to select measurement points on a gold substrate that produce the same reflected SE spectra arises. This question is fundamentally important to be able to implement the virtual substrate method with high precision, and also, to an extent, determine whether or not the virtual substrate measurement requires certain skills, which directly affects the generality of this approach.

To demonstrate the generality and robustness of the procedure to select measurement points, one early prototype sample with marked carbon contamination is used here. The carbon contamination originates from residual organic glue when transferring the graphene flakes from PDMS to the gold substrate. The residual glue is charred by the incident electron beam during measurement. Such carbon contamination is observed in the SEM image in Supplementary Fig. 9 as darker patches on both the gold surface and graphene.

Besides these darker patches (< 50 nm), larger dark and bright areas (~1 μm) appear alternately on the bare gold substrate, which represent different crystallographic orientations of the gold grains. These dark and bright areas can also be found in the graphene region; however, their contrast is lower because of the attenuation by the covering graphene layer. We arranged these gold grains according to their intensities in the SEM image. This order is the same after transfer of the graphene layer onto them even though all the intensities of these gold grains become lower because of the attenuation by the covering graphene layer. That is, the gold grain that shows the strongest intensity in the SEM image remains the strongest after transfer of the graphene layer onto the substrate even though its intensity decreases. Therefore, it is reasonable to think that the bright areas or dark areas

observed in the gold substrate and graphene-covered regions that share the same ranking of intensities should be formed by similar types of gold grains that can produce similar reflected SE spectra after removing the covering graphene layer. The ranking of gold grains can be easily conjectured according to the proportion of gold grains in the region with higher or lower intensities regardless of whether the region is bare gold substrate or covered with graphene. To estimate the proportions of different types of gold grains from the intensities of the SEM image, the renormalization procedure will be helpful to lower the artificial factor in the selection of measurement points in a virtual substrate measurement.

It should be noticed that common SEM images can also be used as a reference to select the measurement points; however, the monochromatic SEM image formed by the electrons with a given energy is generally better to guarantee the reproducibility of one virtual substrate measurement with different experimental conditions. Electrons with energy larger than 10 eV are generally used to form the monochromatic SEM image because they usually show better consistency with different experimental instruments as such electrons are less sensitive to electromagnetic interference than those with lower energies. Furthermore, monochromatic SEM images with a series of energies can be used separately. The renormalized SEM image formed by 10 ± 2 eV electrons is used in the present example. The intensities of the SEM image are renormalized to give the image in Supplementary Fig. 10a.

The intensities of the pixels in the original SEM image are divided into 14 different levels; in other words, the gold grains in both the bare gold substrate and graphene-covered regions are ranked into 14 grades according to their intensities in the SEM image. Furthermore, the renormalization procedure is accompanied with a smoothing operation in which the intensity of one pixel is

averaged together with the intensities of its 24 nearest-neighbour pixels with equal weight. As shown in Supplementary Fig. 10b, the smoothed, renormalized SEM image is more convenient for both the experiment operator and image recognition program to judge the rankings of the gold grains. Considering the attenuation effect of the covering graphene layer, the distribution of the gold grains in the bare gold substrate and graphene-covered regions should be different; generally, the former is broader than the latter. Therefore, we should rank these gold grains in their own regions to guarantee the gold grains that share the same ranking in different regions have the same reflected SE spectra if the covering graphene layer is removed. In that case, the renormalization procedure should be performed separately in bare gold substrate and graphene-covered regions. The resulting renormalized SEM image for bare gold substrate and graphene-covered regions are shown in Supplementary Fig. 10c and d, respectively. Based on these two new renormalized SEM images for different regions, the measurement points can be easily determined by choosing the same ranking of gold grains in different regions. This process to select measurement points can be performed semi-automatically using custom-made software to automatically provide the coordinates of potential measurement points. To select measurement points quickly and easily in the virtual substrate method, some parameters of this custom-made software, such as the level of the renormalization procedure, the degree of smoothing of the image and the threshold value in the image recognition program can be modified according to the performance of the individual experimental instrument. Usually, multiple “monochromatic” SEM images are used together to further verify the selected measurement points.

For some specific cases requiring high precision like measuring the elastic transmission of mono- and bilayer graphene, a pre-testing procedure to evaluate the selected measurement points on the bare substrate before transfer or growth of a nano-overlayer is necessary to increase the precision

of the virtual substrate measurement. The pre-testing procedure involves measuring the reflected SE spectra at the determined measurement points, directly comparing the produced spectra, and then evaluating this group of measurement points by the consistency of the obtained spectra. It should be noted that the pre-testing of measurement points can also be done after virtual substrate measurement by removing the covering graphene layer; however, this destroys the sample. Supplementary Fig. 11 illustrates four pre-testing procedures performed independently on three different samples.

The elastic transmission measurement of mono- and bilayer graphene in the present work was achieved using Sample 1. The pre-testing procedure revealed that the selected measurement points in Sample 1 show the most consistent raw spectra compared with measurement points selected in other samples. With the help of landmarks (easily identifiable precipitates, dust particles or pinholes) on this substrate, we can easily measure these selected measurement points again after transfer or growth of graphene on the substrate (carbon contamination introduced during measurement in the pre-testing procedure was removed by Ar ion sputtering). Usually, excess measurement points will be selected as backups in case wrinkles in the graphene layer happen to block the selected measurement points.

After the pre-testing procedure for the selected measurement points, the graphene layer is transferred to Sample1 (SEM1), as shown in Supplementary Fig. 12a.

The appointed areas for selecting measurement points in the bare gold substrate region (red dashed line) and graphene-covered region (blue dashed line) should be of similar size to minimize the influence from different sample size (different number of candidate gold grains). To further demonstrate the reliability of these selected measurement points, measurement points are also selected on the bare gold substrate (SEM2) about 10 μm away from the boundary of the graphene-

covered region (SEM1) as a control group, as illustrated in Supplementary Fig. 12b. The measurement points on these two different SEM images are determined using the same standards with the help of our custom-made software. Simple schematic diagrams (black and white SEM images with different threshold values) like those shown in Supplementary Fig. 12c and d are used here to briefly demonstrate the process of selecting measurement points on a substrate surface with or without a covering graphene layer. However, these diagrams are not the intermediate product obtained using this custom-made software. Realistic intermediate products like the assisted images obtained by our custom-made software in the process used to select measurement points are similar to the images in Supplementary Fig. 10c and d rather than these black and white SEM images.

Raw spectra and corresponding difference spectra were measured at two regions of the same sample about 10 μm apart corresponding to the SEM images in Supplementary Fig. 12a and b. The raw spectra measured at selected points in the two SEM images are shown in Supplementary Fig. 13a. An enlargement of these raw spectra with short energy range is provided in Supplementary Fig. 13b. The raw spectra measured at different points show excellent agreement. The consistency of these raw spectra taken in the SEM1 region with a smaller sample space (comparable with the sample space of a graphene layer) is a little poorer than that of the spectra measured in the SEM2 region. Small deviations of these raw spectra measured at different sample regions (SEM1 and SEM2) are found, which may originate from sample inhomogeneity, the stability of the instrument changing over time, or both. Such deviation of raw spectra is clearer after averaging the eight spectra measured at different points, as illustrated in Supplementary Fig. 13c. Based on these raw spectra, all 64 possible difference spectra were calculated and are plotted together in Supplementary Fig. 13d and e corresponding to the measurements in region SEM1 and SEM2, respectively. The averaged difference spectra with error bars are shown in Supplementary Fig. 13f. It is interesting

that although there are small but clear deviations in raw spectra obtained at the two different sample surface regions (SEM1 and SEM2), these deviations disappear in the difference spectra, especially in the low energy range below 300 eV. This means these deviations will disappear in the final results of virtual substrate measurement. This result indicates that the virtual substrate method is more robust to sample inhomogeneity and the stability of the instrument changing over time than expected. Of course, to improve virtual substrate measurement, four types of spectra (S(A), S(B), N(A) and N(B)) should be measured within as small a space range and short a time range as possible to avoid the potential sample inhomogeneity and instrument instability. Therefore, short-term repeated measurements for multiple cycles with micrometre distances between different measurement points are the best choice for virtual substrate measurement.

Supplementary Note 3. Performing virtual substrate measurements by scanning SEM image.

The reason why we need to select measurement points before performing a virtual substrate measurement is to verify the identity of reflected SE spectra produced at two determined measurement points like S(A) and N(A). Take a polycrystalline gold substrate for instance, as shown in Supplementary Fig. 14.

It is well known that a covering graphene layer does not affect the relative order of the intensities of gold grains. Therefore, two gold grains that share the same ranking as bare and graphene-covered gold substrates have the potential to produce the same reflected SE spectra after removing the covering graphene layer. Breaking our old pattern of thinking, the pixels of a SEM image formed by detected electrons at a given energy (Supplementary Fig. 14a) can be considered as pixel-sized "fictitious grains". It should be noted that we do not expect that the intensities of these pixel-sized "fictitious grains" represent the behavior of whole spectra at all energies, which is expected when using realistic gold grains. In this case, the pixel-sized "fictitious grains" that share the same ranking of intensity distribution of pixels in the bare gold substrate and graphene-covered regions can be used to perform virtual substrate measurements only at this given energy instead of selecting measurement points on realistic gold grains. The intensity distribution of pixels in the bare gold substrate and graphene-covering regions normalized by the total number of involved pixels are plotted in Supplementary Fig. 14b. The distribution of these pixel-sized "fictitious grains" in the bare gold substrate region is broader than that in the graphene-covered region because of the attenuation effect of the covering graphene layer. Likewise, the presence of a graphene layer does not changing the ranking of pixel-sized "fictitious grains". The pixel-sized "fictitious grains" that have the same ranking in the bare gold substrate and graphene-covered regions can be considered as the same pixel-sized "substrate", analogous to selecting the same type of gold grains. For

instance, the pixel-sized "fictitious grains" with rankings of 20% and 80% can be considered as two different substrates, allowing virtual substrate measurements to be performed. In this case, the difference spectra (only one data point at 100 eV) can be written as $\mathbf{T}^{\mathrm{N}}(\mathbf{R}_{80}-\mathbf{R}_{20})J_0$ and $(\mathbf{R}_{80}-\mathbf{R}_{20})J_0$, and the elastic transmission $\mathbf{T}^{\mathrm{N}}$ can be easily determined from the ratio of these two difference spectra, similar to typical substrate variation measurements performed with designed substrates. Supplementary Fig. 14c shows the accumulated proportion of these two distributions to clearly illustrate the essence of this virtual substrate measurement. Any pixel-sized "fictitious grains" that have the same accumulated proportion value in the range of 15%–85% can be considered as useful pixel-sized "fictitious grains". The matrices representing electron–solid interactions of these pixel-sized "fictitious grains" at a given accumulated proportion value can be written as $\mathbf{R}_x$, which represents mapping from the incident spectrum to a new reflected spectrum of a pixel-sized "substrate" whose accumulated proportion value is x (x = 0–100). Therefore, any combination of two pixel-sized "fictitious grains" can be used to perform virtual substrate measurements, which means there should be infinite number of paired difference spectra $\mathbf{T}^{\mathrm{N}}(\mathbf{R}_{x1}-\mathbf{R}_{x2})J_0$ and $(\mathbf{R}_{x1}-\mathbf{R}_{x2})J_0$, where $x1$ and $x2$ can be any number from 0 to 100 according to the levels of accumulated proportion. According to the definition of the virtual substrate method, measurements using different substrates should produce the same results, which means two accumulated proportion values in the bare gold substrate and graphene-covered regions should be linear except for those at levels that are too low (< 15%) or too high (> 85%). Very obvious linear relationships of these accumulated proportion values can be found for both the bare gold substrate and graphene-covered regions and the ratio of the two slopes of the lines of best fit represents $\mathbf{T}^{\mathrm{N}}$ of graphene. It should be noted that this $\mathbf{T}^{\mathrm{N}}$ determined from the ratio of two slopes can be considered as the average value from every possible combinations of $x1$ and $x2$ except those at

levels that are too low (< 15%) or too high (> 85%). Based on this method of SEM image analysis, the determined $\mathbf{T}^{N}$ at 10, 100, 200 and 400 eV were 0.68, 0.74, 0.85 and 0.90, respectively. These values are a little higher than those measured using a clean graphene/gold sample because of the carbon contamination on the present sample. Generally, the carbon contamination from residual organic glue is greater on gold surfaces than graphene because of the strong adsorption ability of gold surfaces, which increases the measured transmission value. This effect cannot be removed in a virtual substrate measurement. However, this example does reveal that any substrate that shows a relatively stable intensity distribution in a SEM image can be used to perform virtual substrate measurements, not only specially prepared polycrystalline or patterned substrates. In fact, almost every common substrate-supported nanomaterial except those on top of single crystals show a relatively stable intensity distribution in SEM images. Therefore, they can all be directly employed to perform virtual substrate measurements in which pure nanomaterial information at a given energy can be extracted without any additional procedures other than obtaining a SEM image.
